# A Small Molecule Inhibitor of Src Family Kinases Promotes Simple Epithelial Differentiation of Human Pluripotent Stem Cells

**DOI:** 10.1371/journal.pone.0060016

**Published:** 2013-03-20

**Authors:** Xiaojun Lian, Joshua Selekman, Xiaoping Bao, Cheston Hsiao, Kexian Zhu, Sean P. Palecek

**Affiliations:** Department of Chemical & Biological Engineering, University of Wisconsin, Madison, Wisconsin, United States of America; Sanford Burnham Medical Research Institute, United States of America

## Abstract

Human pluripotent stem cells (hPSCs) provide unprecedented opportunities to study the earliest stages of human development *in vitro* and have the potential to provide unlimited new sources of cells for regenerative medicine. Although previous studies have reported cytokeratin 14+/p63+ keratinocyte generation from hPSCs, the multipotent progenitors of epithelial lineages have not been described and the developmental pathways regulating epithelial commitment remain largely unknown. Here we report membrane localization of β-catenin during retinoic acid (RA) – induced epithelial differentiation. In addition hPSC treatment with the Src family kinase inhibitor SU6656 modulated β-catenin localization and produced an enriched population of simple epithelial cells under defined culture conditions. SU6656 strongly upregulated expression of cytokeratins 18 and 8 (K18/K8), which are expressed in simple epithelial cells, while repressing expression of the pluripotency gene Oct4. This homogeneous population of K18+K8+Oct4− simple epithelial precursor cells can further differentiate into cells expressing keratinocyte or corneal-specific markers. These enriched hPSC-derived simple epithelial cells may provide a ready source for development and toxicology cell models and may serve as a progenitor for epithelial cell transplantation applications.

## Introduction

During *in vivo* mouse development, the ectodermal cells in the early embryo exhibit a “default” neural fate and during normal development, bone morphogenetic proteins (BMPs) inhibit this fate and instead specify epidermal lineages [1]. Upon receiving the BMP signal, the ectodermal cells will develop into epidermal progenitors [2]. At the early stage of this development, the epidermis derives from cells expressing the cytokeratins K8 and K18. At later stages of development, these cells gain the potential for stratification, marked by the onset of expression of cytokeratins K5 and K14 and the transcription factor p63 [3]. Mechanisms of human epithelial development have not been clearly mapped, in part due to the lack of readily available *in vitro* model systems. Because human pluripotent stem cells (hPSCs), including human embryonic stem cells (hESCs) [4] and induced pluripotent stem cells (iPSCs) [5], [6], can be propagated indefinitely while still retaining the capacity to differentiate into all somatic cell types [7], they are a potentially inexhaustible supply of cells for development studies, diseases modeling and potentially cell therapies [8], [9], [10].

Using cues from developmental processes, our group developed a directed differentiation protocol that guides hESCs toward enriched populations of keratinocytes using BMP4 and retinoic acid treatment [3]. Application of retinoic acid (RA) and BMP4 has also been effective for keratinocyte differentiation in normal human iPSCs and recessive dystrophic epidermolysis bullosa disease iPSC lines [11]. The keratinocytes generated by this protocol undergo epithelial morphogenesis in engineered tissue constructs [12]. The proper function of the RA pathway during embryonic development may require its communication with other signaling pathways. For example, cross-talk between RA and Wnt signaling is involved in the proliferation of human keratinocytes [13]. RA was shown to suppress the expression of canonical Wnt-dependent genes through direct interaction between RA receptor and β-catenin [14]. Interestingly, another keratinocyte directed differentiation protocol treats hPSCs with BMP4 and ascorbic acid instead of retinoic acid [15]. The effectiveness of retinoic acid or ascorbic acid might be due to different basal media used in these two protocols. Nevertheless both of these protocols relied on exogenous or endogenous BMP signals, consistent with the mechanism of *in vivo* epidermal development. Although these previous studies have reported K14+/p63+ keratinocytes from hESCs and iPSCs, the multipotent progenitors of epithelial lineages have not been isolated and the developmental signaling pathways regulating epithelial commitment still remain largely unknown.

The Src family kinases (SFKs), a family of non-receptor tyrosine kinases that interact with a variety of cellular cytosolic, nuclear, and membrane proteins, play key roles in regulating signal transduction in response to variety of cellular environments. All SFKs are negatively regulated by c-src tyrosine kinase (Csk) and this regulation is indispensable during mouse embryonic development *in vivo*, as Csk-deficient mouse embryos were developmentally arrested at the 10 to 12 somite stage and exhibited growth retardation and necrosis in the neural tissues [16]. The SFK member c-Yes has been implicated in activating self-renewal of mouse embryonic stem cells (mESCs) because knockdown of c-Yes with silencing RNAs led to differentiation [17]. Another SFK member, c-Src, enhances differentiation to primitive ectoderm in mESCs [18]. Therefore, individual SFKs may control distinct and potentially opposing pathways in pluripotent cell self-renewal and differentiation. In humans, there are 11 SFKs which regulate diverse cellular processes including proliferation, adhesion, differentiation, and survival [19]. Activation of SFKs by FGF-2 has been shown to be important for self-renewal of hESCs [20]. Conversely, stage-specific inhibition of SFK signaling has been shown to enhance differentiation of insulin-producing β-cells from hPSCs [21]. The stage-specific roles of SFK signaling on hPSC differentiation to other lineages, especially the necessity and sufficiency of these signals in context of epithelial differentiation, still remain largely unknown. Here, we illustrate that appropriate temporal regulation of SFK signaling via small molecule inhibitors is sufficient to efficiently drive multiple hPSC lines to differentiate to epithelial cells. We then used this method to develop a robust, defined, growth factor-free method of producing simple epithelial cells from hPSCs solely by small molecule-mediated inhibition of SFK signaling.

## Results

### β-catenin Expression Remained Constant during Retinoic Acid Induced Epithelial Differentiation

**Figure 1:**
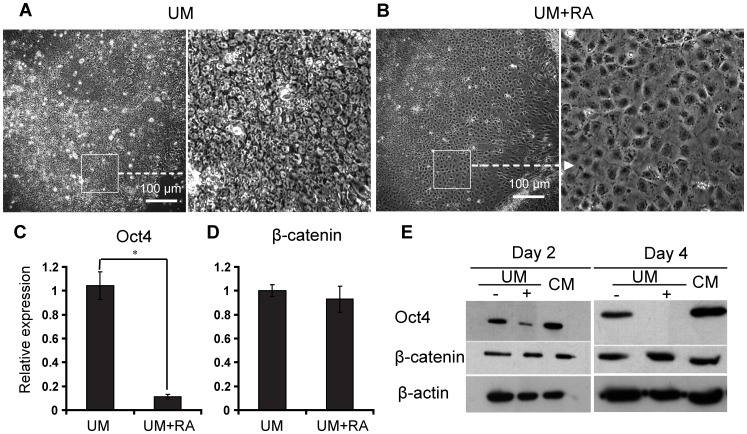
β-catenin expression remains constant during RA-induced epithelial differentiation. (A–B) H9 cells were treated without (A) or with (B) 1 µM RA in UM for 4 days. Representative phase contrast images are shown. Scale bar = 100 µm. (C–D) Quantitative polymerase chain reaction (PCR) of cDNA harvested from H9 cells cultured in UM with or without RA for 4 days. (C): Oct4 expression was significantly reduced upon RA addition; *p<0.05, UM versus RA. (D): β-catenin mRNA expression remained constant upon RA addition, p>0.05, UM versus RA. Error bars represent SEM of three biological replicates. (E) Western blot of protein extracts from H9 hESCs cultured for 4 days in UM without (−) or with (+) RA and CM.

Wnt signaling also has been reported to play key roles in epithelial differentiation and patterning [22], [23], [24]. In order to test if Wnt signaling is involved in RA-induced epithelial differentiation of hESCs, we treated monolayer-cultured H9 hESCs with DMSO or RA in unconditioned medium (UM). Addition of RA induced the formation of cobblestone outgrowths, whereas control cells retained a more compact morphology (**Fig. 1A, 1B**). Quantitative RT-PCR analysis demonstrated a pronounced decrease in expression of Oct4, a pluripotency marker, 4 days after RA treatment (**Fig. 1C** **)**. Transcription of β-catenin, however, was not significantly affected by the addition of RA (**Fig. 1D**). H9 hESCs exhibited diminished Oct4 protein expression after 4 days of RA treatment based on western blot analysis, whereas DMSO-treated control cultures in UM and cells cultured in mouse embryonic fibroblast-conditioned medium (CM) still expressed high levels of Oct4 (**Fig. 1E**). The total amount of β-catenin protein did not change substantially across RA-treated, DMSO-treated, and CM-cultured cells (**Fig. 1E**).

### RA-induced Epithelial Differentiation Correlates with β-catenin Membrane Localization and Downregulation of Canonical Wnt Signaling

**Figure 2:**
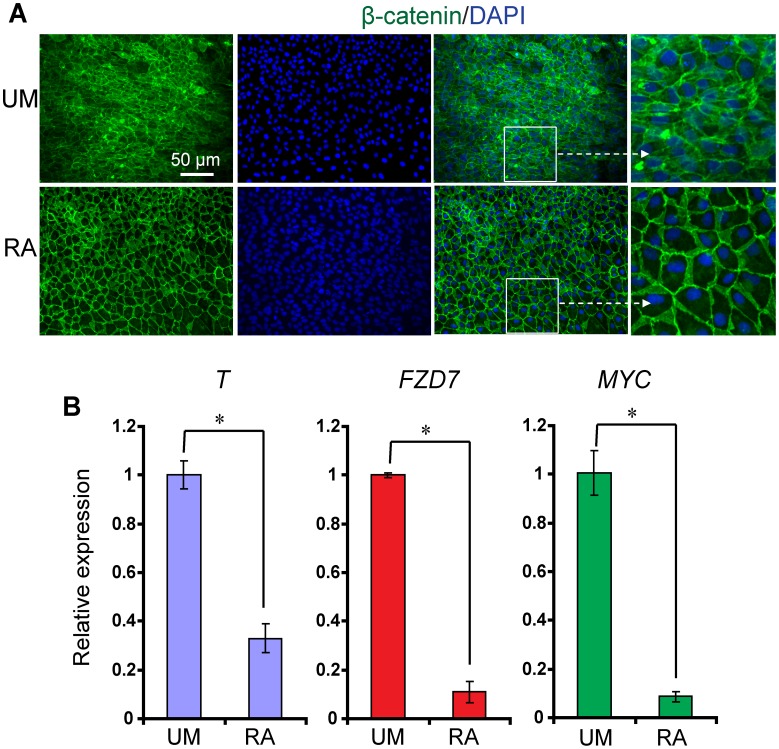
RA-induced epithelial differentiation correlates with β-catenin membrane localization. (A) Representative β-catenin (green) immunocytochemistry and Hoechst (blue) staining of H9 hESCs cultured without or with RA in UM for 4 days. (B) Quantitative polymerase chain reaction (PCR) of cDNA harvested from H9 cells cultured in UM with or without RA for 4 days. Expression of Wnt signaling target genes *T*, *FZD7* and *MYC* was significantly reduced upon RA addition; *p<0.05, UM versus RA. Error bars represent SEM of three biological replicates.

β-catenin is a key component in canonical Wnt pathway, acting as a transcriptional transactivator for canonical Wnt signaling activation [25], [26], [27]. In addition, β-catenin has additional roles at the cell membrane mediating cell-cell adhesion as a component of the adherens junction [28]. The importance of β-catenin translocation between the nucleus and the membrane in the context of epithelial commitment is unclear. To determine whether β-catenin localization changes during RA-induced epithelial differentiation, we immunostained RA- and DMSO-treated H9 hESCs with an anti-β-catenin antibody. Whereas DMSO-treated control cells showed membrane, cytoplasmic, and nuclear localization of β-catenin, RA-treated cells exhibited uniform β-catenin localization to the cell membrane but very little β-catenin in the nucleus (**Fig. 2A**). Since the total amount of β-catenin remained constant (**Fig. 1E**), RA-induced membrane translocation of β-catenin might be expected to downregulate canonical Wnt signaling. To determine whether β-catenin translocation to the membrane in RA-treated cells efficiently inhibited the transcription of endogenous TCF/LEF-responsive genes, we assessed transcript levels of Wnt target genes, Brachyury (*T*) [29], [30], Frizzled family receptor 7 (*FZD7*) [31] and c-myc (*MYC*) [32]. Wnt target genes substantially downregulated upon RA treatment as compared to control cultures (**Fig. 2B**). Overall, these results indicate a correlation between β-catenin localization to the membrane and epithelial differentiation in hESCs.

### Retinoic Acid Induces E-cadherin Upregulation during Epithelial Differentiation

**Figure 3:**
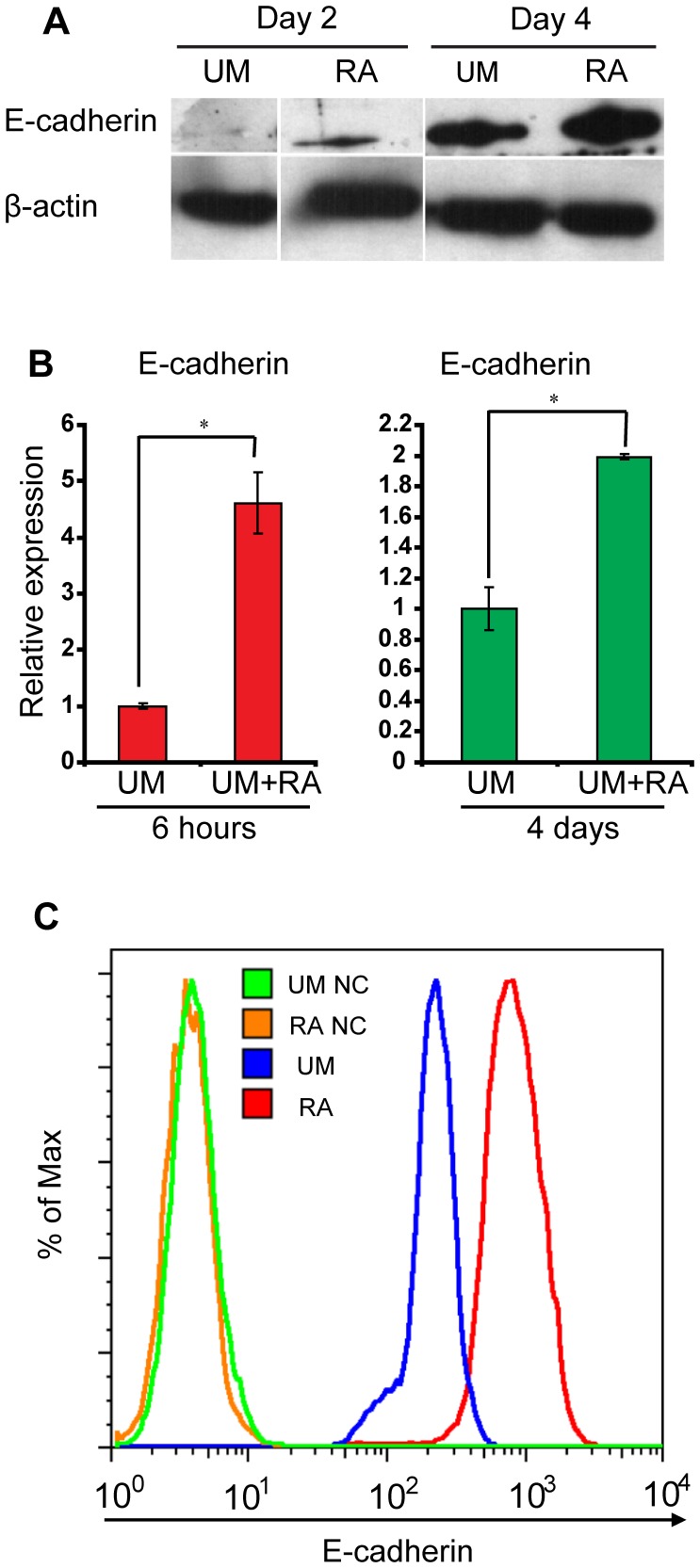
Retinoic acid induces E-cadherin upregulation during epithelial differentiation. (A) Western blot of protein extracts from H9 hESCs cultured for 4 days in UM without or with RA. E-cadherin protein expression increased in the presence of RA. (B) Quantitative polymerase chain reaction (qPCR) of cDNA harvested from H9 cells cultured in UM with or without RA for 6 hours or 4 days. mRNA of E-cadherin increased by 4-fold or 2-fold in RA-treated cells after 6 hours or 4 days respectively; *p<0.05, UM versus RA. (C) H9 hESCs were cultured for 4 days in UM without or with RA. Flow cytometry with a FITC-conjugated monoclonal E-cadherin antibody was performed. “UM NC” and “RA NC” indicate negative isotype control samples for cells cultured in UM and UM+RA respectively.

E-cadherin is crucial for pluripotent stem cell survival and pluripotency [33], [34]. Previous research demonstrated that E-cadherin binding prevents β-catenin nuclear localization and canonical Wnt signaling activation [35]. In order to test the hypothesis that increased β-catenin membrane localization in RA-treated cells is a result of elevated E-cadherin expression, we compared E-cadherin protein in RA and DMSO-treated hESCs via western blot. Indeed, more E-cadherin protein was expressed in RA-treated samples than in DMSO-treated controls at 2 and 4 days following RA treatment (**Fig. 3A**). 6 hours after RA treatment, mRNA of E-cadherin increased by 4-fold in RA-treated cells (**Fig. 3B**). 4 days after treatment, RA-treated cells still showed a 2-fold greater expression of E-cadherin than untreated cells (**Fig. 3B**). In order to quantify the intensity of E-cadherin expression per cell, the cells cultured with or without RA treatment for 4 days were singularized and assayed via flow cytometry with a FITC-conjugated monoclonal E-cadherin antibody. The average fluorescence intensity of E-cadherin per cell in RA-treated cells was greater than E-cadherin expression in control cells and the entire population exhibited an upregulation of E-cadherin as a result of RA treatment. Representative histograms of E-cadherin expression are shown in **Fig. 3C**. These results suggest that increased E-cadherin expression induced by RA might sequester more β-catenin to the cell membrane.

### Modulation of β-catenin Localization Promotes Highly Efficient Epithelial Differentiation

β-catenin localization within the cell is suggestive of its function [36]. Membrane localized β-catenin likely mediates cadherin linkages with the cytoskeleton at adherens junctions while nuclear localized β-catenin interacts with co-factors to initiate transcription of genes regulated by canonical Wnt signaling [25]. For example, translocation of β-catenin from the membrane to the nucleus occurs during the epithelial-mesenchymal transition (EMT), which is essential for organ development in the embryo [37]. However, the potential role of β-catenin translocation on epithelial differentiation of hPSCs is unknown. This switch from membrane to nuclear localization of β-catenin can be regulated by receptor tyrosine kinases (e.g. EGF receptors) and cytoplasmic tyrosine kinases (e.g. Src family kinases) which phosphorylate specific tyrosine residues (Y654) of β-catenin leading to dissociation of the cadherin-catenin complex. Then the β-catenin is free to localize to the nucleus [38].

**Figure 4:**
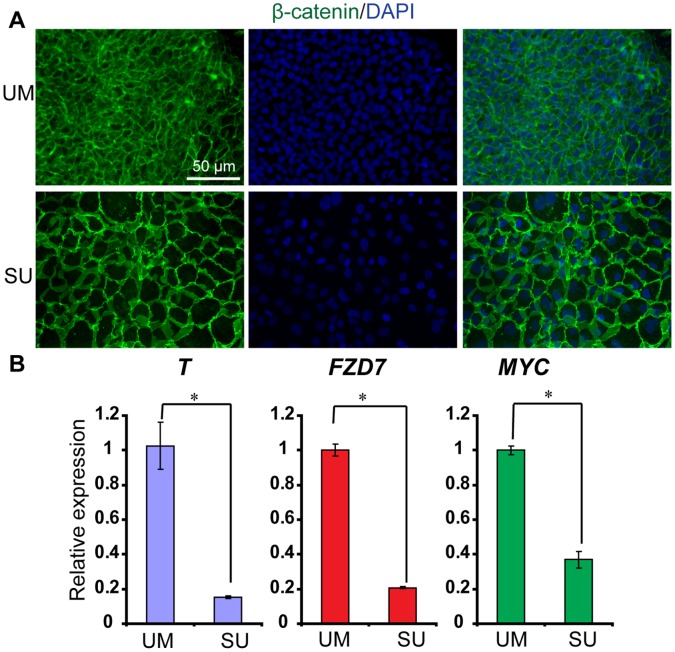
The SFK inhibitor SU modulates β-catenin intracellular localization. (A) Representative Oct4 (red) and β-catenin (green) immunocytochemistry and Hoechst (blue) staining of H9 hESCs cultured without or with SU in UM for 4 days. (B) Quantitative polymerase chain reaction (PCR) of cDNA harvested from H9 cells cultured in UM with or without SU for 4 days. Expression of Wnt target genes *T*, *FZD7,* and *MYC* was significantly reduced upon RA addition; *p<0.05, UM versus RA. Error bars represent SEM of three biological replicates.

We reasoned that a SFK inhibitor, which prevents phosphorylation of β-catenin tyrosine residues, might block the dissociation of the cadherin-catenin complex and increase the extent of membrane localization of β-catenin, thus inducing epithelial differentiation of hPSCs. To probe the effects of SFK inhibitor on the β-catenin distribution with the cell, we treated undifferentiated H9 hESCs with the SFK inhibitor SU6656 (SU). Clear membrane β-catenin localization was observed in SU6656-treated sample when compared to control samples (**Fig. 4A**). Furthermore, expression of Wnt target genes (*T*, *FZD7* and *MYC*) was highly downregulated upon SU6656 treatment as compared to control cultures (**Fig. 4B**).

**Figure 5:**
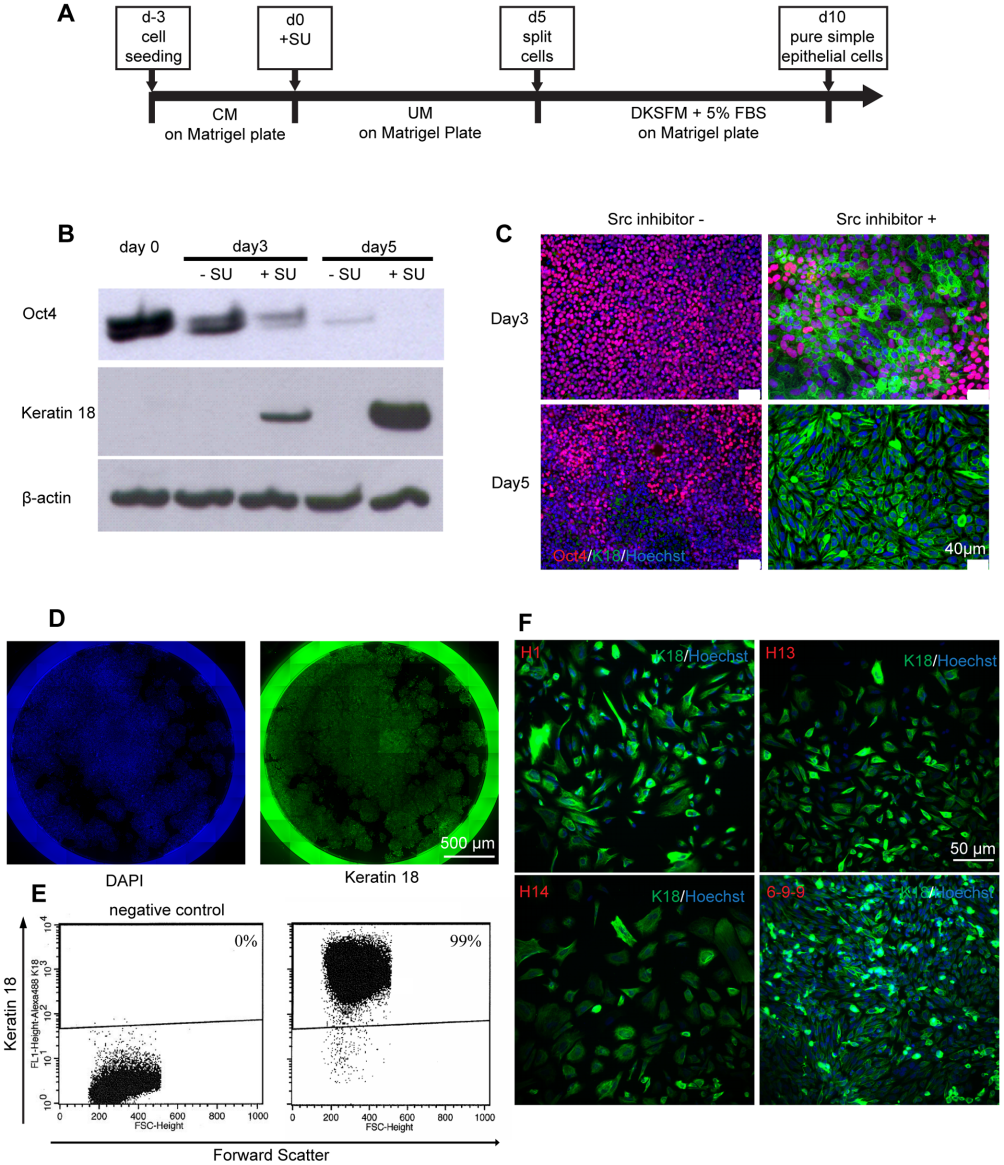
Modulation of β-catenin localization promotes highly efficient epithelial differentiation. (A) Schematic of the protocol for differentiation of hPSCs to simple epithelial cells via treatment with the SFK inhibitor SU6656. (B) Western blot of protein extracts from H9 hESCs cultured for 5 days in UM without or with SU. Cytokeratin K18 protein expression was only detected in SU-treated samples. (C) Representative Oct4 (red) and K18 (green) immunocytochemistry and Hoechst (blue) staining of H9 hESCs cultured without or with SU in UM for 5 days. (D) Whole-well imaging of K18 (green) and Hoechst (blue) stained H9 cells cultured in a 12 well plate containing UM with SU for 5 days. (E) K18 flow cytometry of H9 hESCs differentiated 5 days in UM with SU. The left panel shows the negative control sample treated with the isotype control antibody. (F) Representative K18 (green) immunocytochemistry and Hoechst (blue) staining images of hESCs (H1, H13 and H14) and iPSCs (6-9-9) cultured in UM with SU for 5 days.

To investigate the effects of SFKs on epithelial differentiation of hPSCs, we treated H9 hESCs with SU and monitored the expression of Oct4 and K18 (**Fig. 5A**). Consistent with previous findings [20], [39], the pluripotent protein marker Oct4 downregulated more rapidly in the SU-treated samples than in the DMSO-treated control. In the meantime, the simple epithelial protein K18 was only detected in the SU-treated sample, with elevated levels at day 5 compared to day 3, indicating epithelial differentiation in the SU-treated sample (**Fig. 5B**). Immunostaining of Oct4 and K18 in the samples treated with or without SU revealed that SFK inhibition gradually increased K18 expression while downregulating Oct4 expression (**Fig. 5C**). Whole-well imaging demonstrated uniform K18 expression on day 5 in SU-treated hESCs (**Fig. 5D**). In addition, flow cytometry analysis demonstrated that virtually all cells in the SU-treated population expressed K18 at day 5 (**Fig. 5E**). SFK inhibitor treatment also efficiently induced epithelial differentiation, defined by K18 expression, in other hPSC lines, including H1, H13, and H14 hESCs and 6-9-9 iPSCs (**Fig. 5F**). Another tyrosine kinase inhibitor, AG825, similarly induced epithelial differentiation (**Fig. S1**).

**Figure 6:**
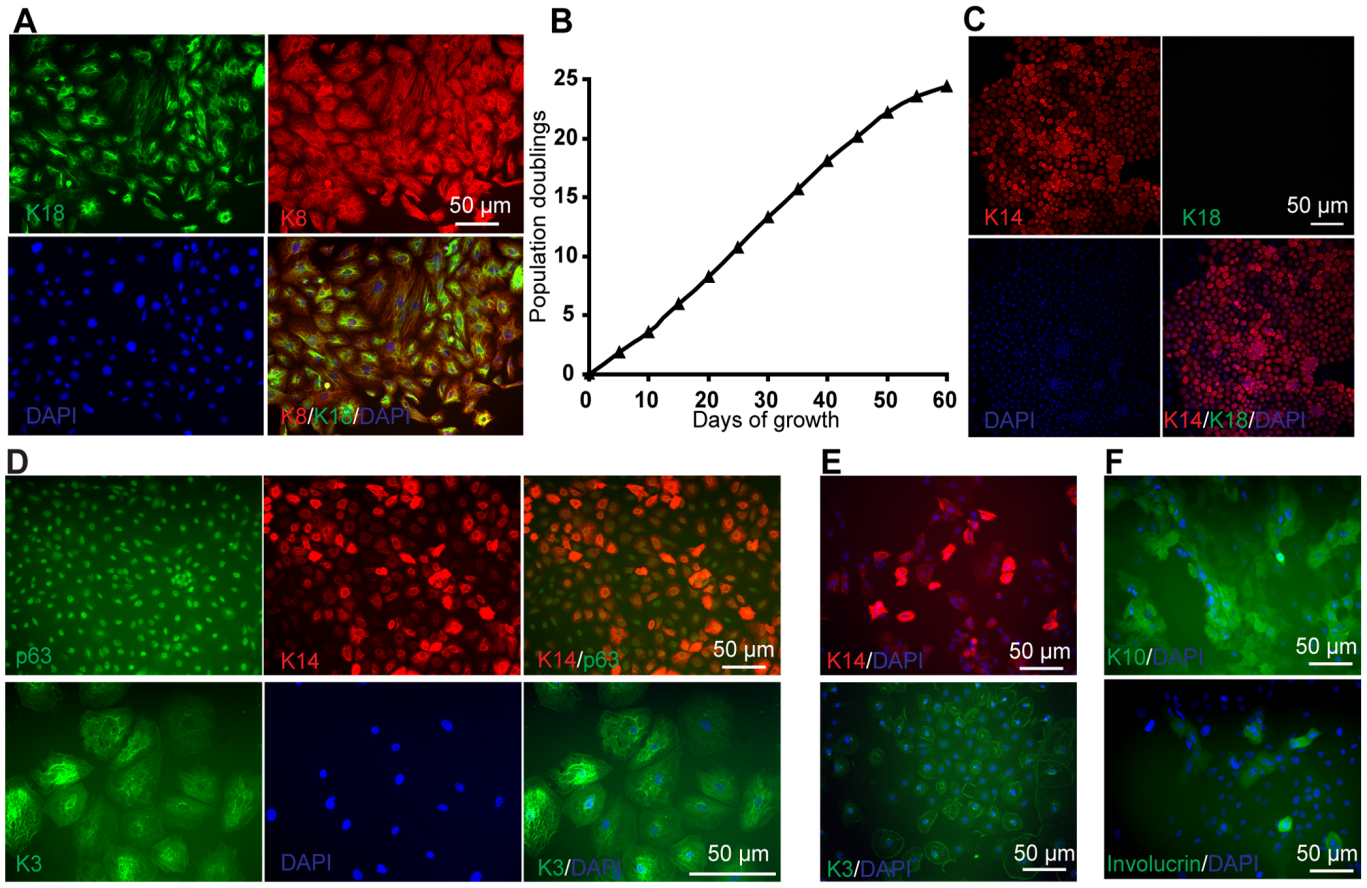
Expansion and terminal differentiation potential of simple epithelial cells from hPSCs. (A) H9 cells were treated with SU for 5 days in UM. Representative K18 (green) and K8 (red) immunocytochemistry and DAPI (blue) staining are shown. (B) Subculture of H9 derived simple epithelial cells (K18+K8+) over multiple passages on 0.1% gelatin-coated 6-well plates. (C–E) The H9-derived simple epithelial (K18+/K8+) cells were treated with 1 µM RA and 10 ng/ml BMP4 for 4 days and then cultured in DKSFM for 10 days. Immunostaining of (C) K18 (green) and K14 (red), (D) p63 (green) and K14 (red), K3 (green) were performed. (E) H9-derived K18+ cells were Accutase treated to generate single cells and plated on Matrigel-coated six-well plates in DKSFM with 5% FBS at a density of 5,000 cells per well. Cells were cultured with medium changes every 7 days for 2 weeks. The resulting clones were manually picked and plated on Matrigel-coated 24-well plates at a density of one clone per well. Cells were treated with 1 µM RA for 4 days and then cultured with medium changes every 3 days for another 2 weeks. At day 30, cells were fixed and stained with K14 (red) and K3 (green) antibodies. Scale bars = 50 µm. (F) Simple epithelial cell derived K14+ cells were cultured in DKSFM supplemented with 1 mM Ca^2+^ for 4 days. Immunostaining of K10 (green) and Involucrin (green) were performed. Scale bars = 50 µm.

We next examined the multipotency of K18-expressing cells differentiated from hESCs via treatment with a SFK inhibitor. H9 cells were first differentiated for 5 days in the presence of SU and then replated on coverslips for imaging. These H9-derived simple epithelial cells not only expressed K18, but also Keratin 8 (K8) (**Fig. 6A**). Similar results have been observed in K18+ cells differentiated from the 19-9-11 iPSC line (**Fig. S2A**). These simple epithelial cells (K18+/K8+) exhibited a high proliferation capacity, demonstrated by 24 population doublings over 60 days (**Fig. 6B**). After 60 days expansion, these cells still express K18 (**Fig. S3**). We then examined the capacity of hPSC-derived simple epithelial cells to form keratinocytes upon treatment with RA and BMP4. K18+ cells were treated with 1 µM RA and 10 ng/ml BMP4 for 4 days and then cultured in defined keratinocyte serum free medium for another 10 days. Greater than 90% K14+ cells were generated with this protocol (**Fig. S2B**)**.** These keratinocytes showed a pronounced cobblestone-like morphology (**Fig. S2C**) and expressed K14 and p63, but not K18 (**Fig. 6C and D**). Interestingly, we also observed about 5% of cells expressing the corneal epithelial marker K3 (**Fig. 6D** and **Fig. S2D**). Clonal analysis revealed that the differentiated populations derived from single K18+ cells contained both K14+ cells and K3+ cells (**Fig. 6E** **, Fig. S4**). Moreover, after culturing differentiated K14+ cells in high calcium medium (1 mM Ca^2+^) to induce *in vitro* epidermal differentiation, they expressed Keratin 10 (K10) and involucrin (**Fig. 6F**), markers of terminal epidermal differentiation, indicating that these simple epithelial-derived cells possess the capacity for epidermal differentiation.

## Discussion

In the mouse, the crucial *in vivo* role of p63 during keratinocyte specification has been identified as a result of defects in the epidermis and epithelial appendages upon knocking out p63 [40]. p63 also has been shown to play key roles during *in vitro* epithelial differentiation of both mouse and human pluripotent stem cells [3], [41], [42], [43]. RA has been shown to induce p63 expression in keratinocytes [44]. We have previously demonstrated that treatment of undifferentiated hESCs with RA in the presence of endogenous/exogenous BMP signaling is an effective means of producing high-purity populations of K14+/p63+ keratinocytes [3]. Though the important molecular mechanisms underlying keratinocyte specification from epidermal progenitors have been identified, less is known about the mechanisms regulating development of epithelial progenitors with the capacity to terminally differentiate to epidermal keratinocytes.

In this study, we found that RA signaling induced localization of β-catenin to the cell membrane, diminished the amount of nuclear β-catenin, and downregulated canonical Wnt signaling during the first 5 days of hPSC differentiation. These results are consistent with previous reports of suppression of canonical Wnt signaling by RA signaling in human cancer cells [14]. Our results and previous reports suggest that direct regulation of β-catenin-LEF/TCF signaling is one mechanism whereby RA influences development, cell differentiation, and cancer. According to this hypothesis, we reasoned that downregulation of canonical Wnt signaling by other means should also be able to induce simple epithelial differentiation of hPSCs. We choose to manipulate Src family kinase activity because SFKs have been shown to directly phosphorylate β-catenin at Y654 and this phosphorylation inhibits β-catenin binding to E-cadherin [45]. Conversely, Src family kinase inhibitor treatment has been shown to increase β-catenin/E-cadherin colocalization in human cancer cells [46]. Activation of SFKs by FGF-2 has been demonstrated to be important for self-renewal of hESCs [20]. Here, we demonstrate that the Src family kinase inhibitor SU6656 promotes efficient differentiation of hPSCs to K18+/K8+/Oct4− cells. These simple epithelial cells (K18+/K8+/Oct4−) retained the ability to further differentiate to keratinocytes (K14+/p63+/K18− cells) and corneal (K3+ cells) epithelial cells upon treatment with RA and BMP4. When cultured in medium containing 1 mM Ca^2+^ for another 4 days, the keratinocytes underwent epidermal differentiation and expressed the suprabasal markers Keratin 10 and involucrin. These results demonstrate that the simple epithelial cells generated by SU6656 treatment are multipotent and capable of further differentiation upon receiving proper cues. Therefore, the simple epithelial differentiation model we have provided is likely to help understand early developmental changes that might occur during embryonic epithelial development.

## Materials and Methods

### Cell Culture and Differentiation

Transgene free human iPSCs (6-9-9, 19-9-11) [47] and hESCs (H9, H13, H14) [4] were maintained in hESC unconditioned medium (UM): DMEM/F12 culture medium supplemented with 20% KnockOut serum replacer, 0.1 mM non-essential amino acids, 1 mM L-glutamine (all from Invitrogen), 0.1 mM β-mercaptoethanol (Sigma) and 10 ng/ml human bFGF (Invitrogen) on MEF feeder cells. Conditioned medium (CM) is hESC medium conditioned by mouse embryonic fibroblasts (MEFs) for 24 hours [48]. For feeder-free culture, hPSCs were maintained on Matrigel (BD Biosciences) or Synthemax plates (Corning) in mTeSR1 medium (STEMCELL Technologies) and passaged every 4–5 days using Versene (Life Technologies).

For directed simple epithelial differentiation, hPSCs colonies cultured in CM were switched to hESC unconditioned medium (UM) without bFGF and a combination of DMSO (Sigma), SU6656 (Sigma) for 5 days. At this stage, the generated K18+Oct4− cells can be further differentiated into keratinocytes by another 4 days in UM supplemented with 1 µM retinoic acid (Sigma) and 10 ng/ml BMP4 (Life Technologies) and the sub-cultured in defined keratinocyte serum free medium (DKSFM, Life Technologies).

### Clonal Analysis of Simple Epithelial Cells

hESC-derived K18+ cells were Accutase (Life technologies) treated for 10 minutes to generate single cells and plated on Matrigel-coated six-well plates (BD Biosciences) in DKSFM with 5% FBS at a density of 5,000 cells per well. Cells were cultured with medium changes every 7 days for 2 weeks. The resulting single K18+ cell-derived clones were manually picked and replated on a Matrigel-coated 24-well plate at a density of one clone per well. Cells were treated with 1 µM RA for 4 days and then cultured with medium changes every 3 days for another 2 weeks before performing immunostaining with K14 and K3 antibodies.

### RT-PCR and Quantitative RT-PCR

Total RNA was prepared with the RNeasy mini kit (QIAGEN) and treated with DNase (QIAGEN). 1 µg RNA was reverse transcribed into cDNA via Oligo (dT) with Superscript III Reverse Transcriptase (Invitrogen). Real-time quantitative PCR was done in triplicate with iQ SYBR Green SuperMix (Bio-Rad). RT-PCR was done with Gotaq Master Mix (Promega) and then subjected to 2% agarose gel electrophoresis. *ACTB* was used as an endogenous control. The primer sequences are listed in Table 1.

**Table 1:** Primers for quantitative RT-PCR.

Genes	Sequences (5′ - 3′)	Size (bp)
*GAPDH*	**F:** GTGGACCTGACCTGCCGTCT **R:** GGAGGAGTGGGTGTCGCTGT	152
*OCT4*	**F:** CAGTGCCCGAAACCCACAC **R:** GGAGACCCAGCAGCCTCAAA	161
*CTNNB1*	**F:** CCCACTAATGTCCAGCGTTT **R:** AACGCATGATAGCGTGTCTG	217
*T*	**F:** AAGAAGGAAATGCAGCCTCA **R:** TACTGCAGGTGTGAGCAAGG	101
*FZD7*	**F:** GATGATAACGGCGATGTGA **R:** AACAAAGCAGCCACCGCAGAC	213
*MYC*	**F:** GCGTCCTGGGAAGGGAGATCCGGAGC **R:** TTGAGGGGCATCGTCGCGGGAGGCTG	302
*CDH1*	**F:** AGGAATTCTTGCTTTGCTAATTCTG **R:** CGAAGAAACAGCAAGAGCAGC	50

### Immunofluorescence Assay

Cells were fixed in 4% paraformaldehyde for 15 minutes at room temperature before blocking and permeabilizing with 5% milk in phosphate-buffered saline (PBS) with 0.4% Triton X-100. Primary antibody was incubated overnight at 4°C in blocking buffer, and the sample was then incubated with secondary antibody for 1 hour at room temperature and then stained with Hoechst dye for 5 minutes in PBS. Immunofluorescence images were examined with an epifluorescence microscope (Leica DM IRB) and imaged using QImaging Retiga 4000R camera. Details and concentrations of the antibodies used in this study are provided in Table 2.

**Table 2:** Antibodies used for characterization of hPSCs derived simple epithelial cells.

Antibody	Isotype/Source/cat. no./clone	Concentration
Oct-3/4	mouse IgG2b/Santa Cruz/sc-5279/C-10	1∶100
β-catenin	mouse IgG1/BD bioscience/610154/14	1∶200
E-cadherin-FITC	mouse IgG2a/BD transductiuon/612131/36/E-cadherin	1∶200
p63	mouse IgG2a/Santa Cruz/sc-8431/4A4	1∶100
Cytokeratin K3/K76	mouse IgG1/Millipore/CBL218/AE5	1∶100
Cytokeratin 10	mouse IgG1/Thermo Scientific/MS-611-P0/DE-K10	1∶100
Cytokeratin 8	rabbit IgG/Thermo Scientific/RM-2107-S0/EP1628Y	1∶200
Cytokeratin 14	rabbit IgG/Thermo Scientific/RB-9020-P1	1∶200
Cytokeratin 18	mouse IgG1/Thermo Scientific/MS-142/DC10	1∶200
Filaggrin	goat IgG/Santa Cruz/sc-25896/N-20	1∶200
Involucrin	goat IgG/Santa Cruz/sc-15223/N-17	1∶200
secondary antibody	Alexa 488 Goat anti Ms IgG1/A-21121	1∶1000
secondary antibody	Alexa 488 Goat anti Rb IgG/A-11008	1∶1000
secondary antibody	Alexa 594 Goat anti Ms IgG2b/A-21145	1∶1000
secondary antibody	Alexa 594 Goat anti Rb IgG/A-11012	1∶1000
secondary antibody	Alexa 647 Goat anti Ms IgG2b/A-21242	1∶1000
secondary antibody	Alexa 647 Goat anti Rb IgG/A-21244	1∶1000

### Western Blot Analysis

Cells were lysed in M-PER Mammalian Protein Extraction Reagent (Pierce) in the presence of Halt Protease and Phosphatase Inhibitor Cocktail (Pierce). Proteins were separated by 10% Tris-Glycine SDS/PAGE (Invitrogen) under denaturing conditions and transferred to a nitrocellulose membrane. After blocking with 5% milk in TBST, the membrane was incubated with primary antibody overnight at 4°C. The membrane was then washed, incubated with an anti-mouse/rabbit peroxidase-conjugated secondary antibody (1∶1000, Cell Signaling) at room temperature for 1 hr, and developed by SuperSignal chemiluminescence (Pierce). Antibodies and concentrations are listed in Table 2.

### Flow Cytometry

Single-cell suspensions were prepared using 10 minutes of incubation in accutase (Life Technologies) followed by 20 minutes fixation in 1% paraformaldehyde and 30 minutes permeabilization in 90% methanol on ice. Primary antibodies were incubated overnight in PBS with 0.5% BSA and 0.1% Triton X-100 at 4°C. After a 30 minutes secondary stain at room temperature, cells were acquired on a FACSCalibur using CellQUEST software. Antibodies and concentrations are listed in Table 2.

### Statistics

Data are presented as mean ± standard error of the mean (SEM). Statistical significance was determined by Student’s t-test (two-tail) for two groups. *P*<0.05 was considered statistically significant.

## Supporting Information

Figure S1 **AG825 treatment induces highly efficient epithelial differentiation.** (A) H9 cells were treated without or with 4 µM AG825 in UM and CM for 4 days. Representative phase contrast images are shown. Scale bar = 100 µm. (B) After 4 days of differentiation, cells were fixed and immunostaining of K18 was performed. Representative K18 (green) immunocytochemistry is shown.
(TIF)

Figure S2 **Terminal differentiation potential of simple epithelial cells from hPSCs.** (A) 19-9-11 cells were treated with SU for 5 days in UM. Representative K18 (green) and K8 (red) immunocytochemistry and DAPI (blue) staining are shown. (B–C) The H9-derived simple epithelial cells (K18+/K8+) were treated with 1 µM RA and 10 ng/ml BMP4 for 4 days and then cultured in DKSFM for 10 days. (B) Flow cytometric histograms of K14+ expression in the differentiated cells. The red histogram represents K14 expression and the green histogram is an isotype control. (C) Representative phase contrast image illustrating the morphology of the K14+ cells. (D) Flow cytometric histograms of K3+ expression in the differentiated cells. The red histogram shows K3 expression and the green histogram is an isotype control.
(TIF)

Figure S3 **Keratin 18 expression in simple epithelial cells after two month expansion.** Subculture of H9-derived simple epithelial cells (K18+) over 60 days. After 60 days of expansion, cells were fixed and immunostaining of K18 was performed. Representative K18 (green) immunocytochemistry and DAPI (blue) staining are shown.
(TIF)

Figure S4 **Single K18+ cell differentiation to K14+ and K3+ cells.** Single K18+ cell-derived clones were manually picked and plated on Matrigel-coated 24-well plates at a density of one clone per well. Cells were treated with 1 µM RA for 4 days and then cultured with medium changes every 3 days for another 2 weeks before performing immunostaining with K14 (red) and K3 (green) antibodies. Scale bars = 50 µm.
(TIF)
