# Supplementary figures and images for: A Small Molecule Inhibitor of Src Family Kinases Promotes Simple Epithelial Differentiation of Human Pluripotent Stem Cells
Source: PLoS One. 2013 Mar 20;8(3):e60016. doi: 10.1371/journal.pone.0060016 (PMC3603942; doi:10.1371/journal.pone.0060016)

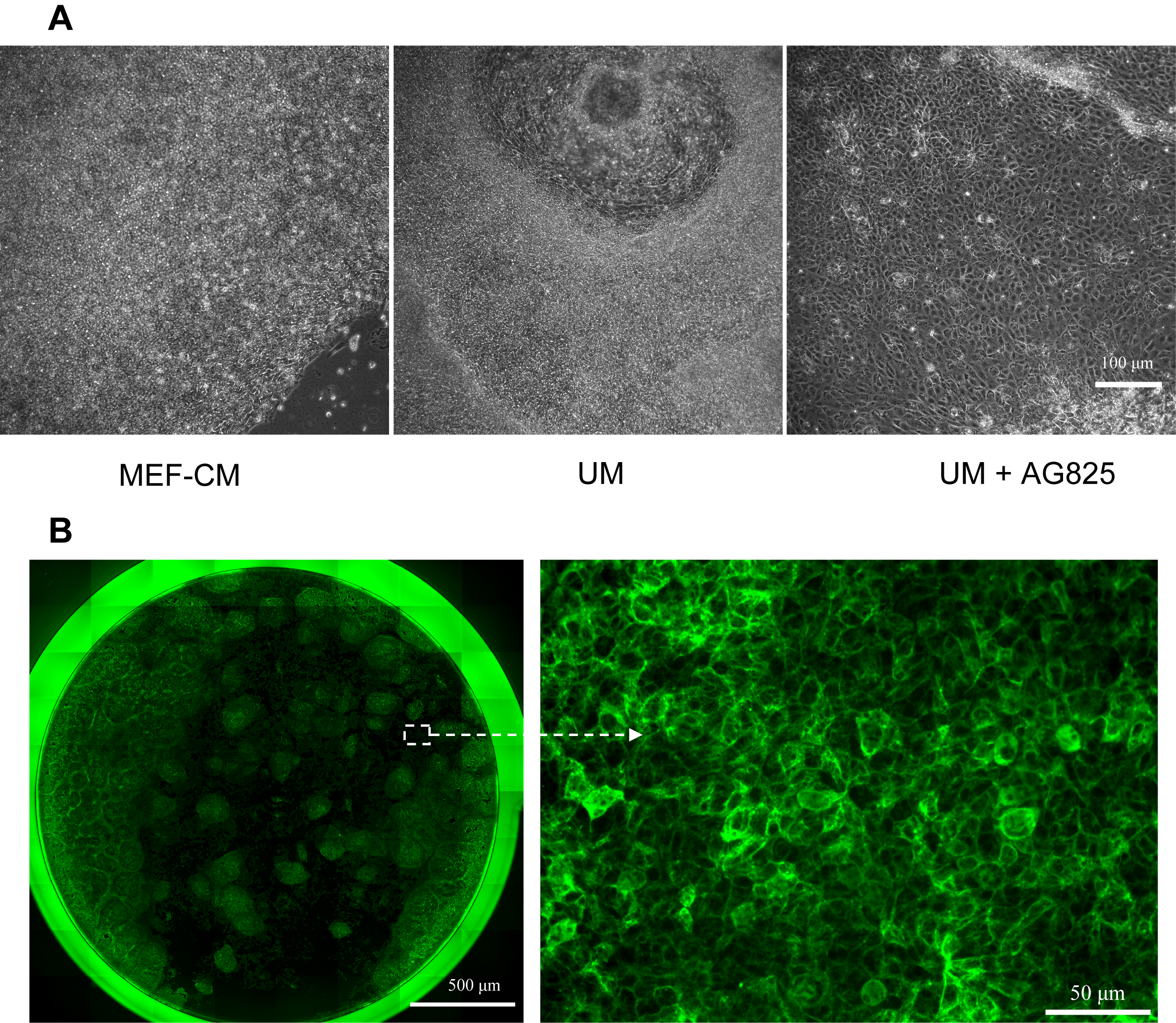

Supplement: Figure S1 — AG825 treatment induces highly efficient epithelial differentiation. (A) H9 cells were treated without or with 4 µM AG825 in UM and CM for 4 days. Representative phase contrast images are shown. Scale bar = 100 µm. (B) After 4 days of differentiation, cells were fixed and immunostaining of K18 was performed. Representative K18 (green) immunocytochemistry is shown. (TIF) [file pone.0060016.s001.tif]

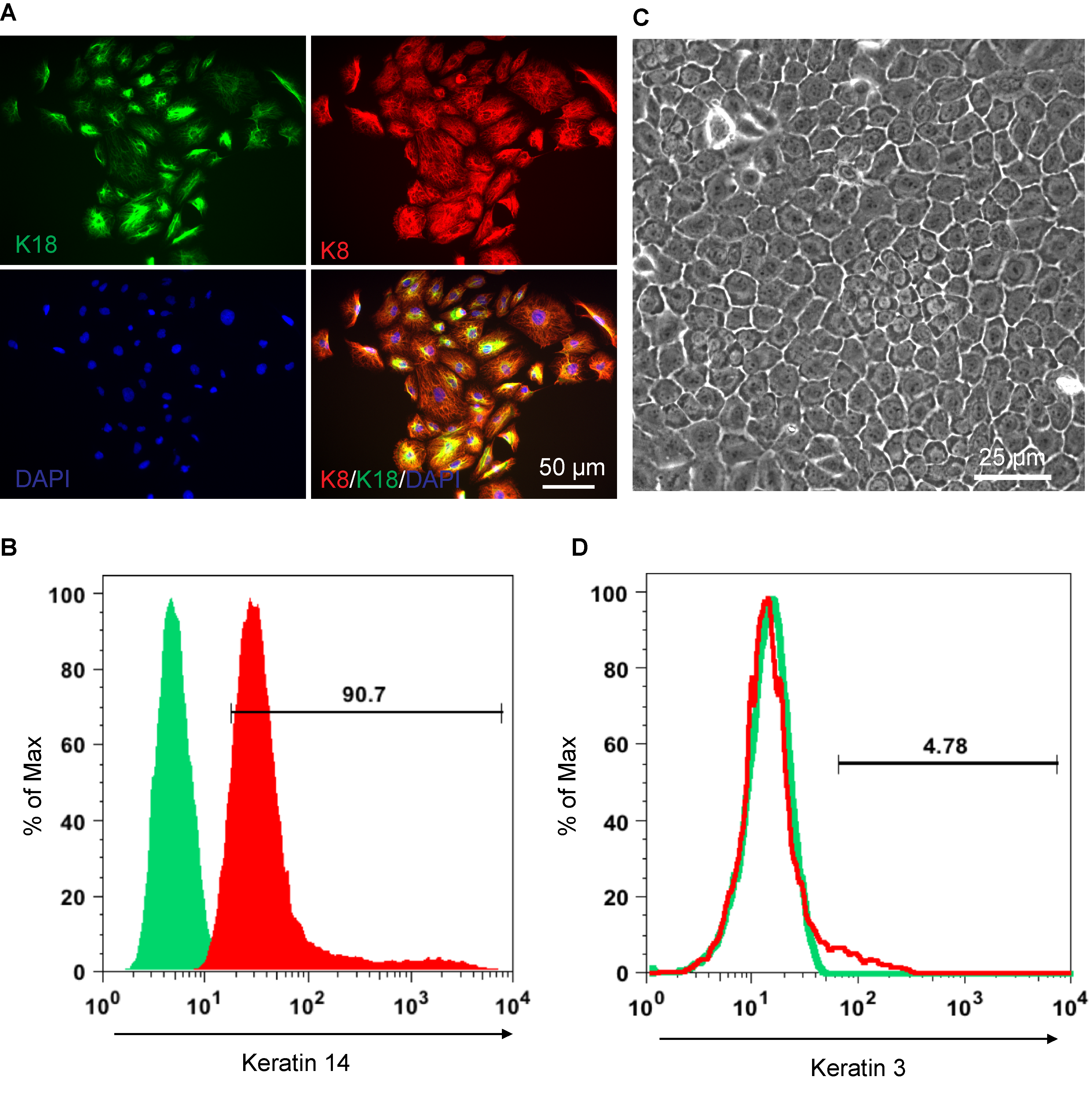

Supplement: Figure S2 — Terminal differentiation potential of simple epithelial cells from hPSCs. (A) 19-9-11 cells were treated with SU for 5 days in UM. Representative K18 (green) and K8 (red) immunocytochemistry and DAPI (blue) staining are shown. (B–C) The H9-derived simple epithelial cells (K18+/K8+) were treated with 1 µM RA and 10 ng/ml BMP4 for 4 days and then cultured in DKSFM for 10 days. (B) Flow cytometric histograms of K14+ expression in the differentiated cells. The red histogram represents K14 expression and the green histogram is an isotype control. (C) Representative phase contrast image illustrating the morphology of the K14+ cells. (D) Flow cytometric histograms of K3+ expression in the differentiated cells. The red histogram shows K3 expression and the green histogram is an isotype control. (TIF) [file pone.0060016.s002.tif]

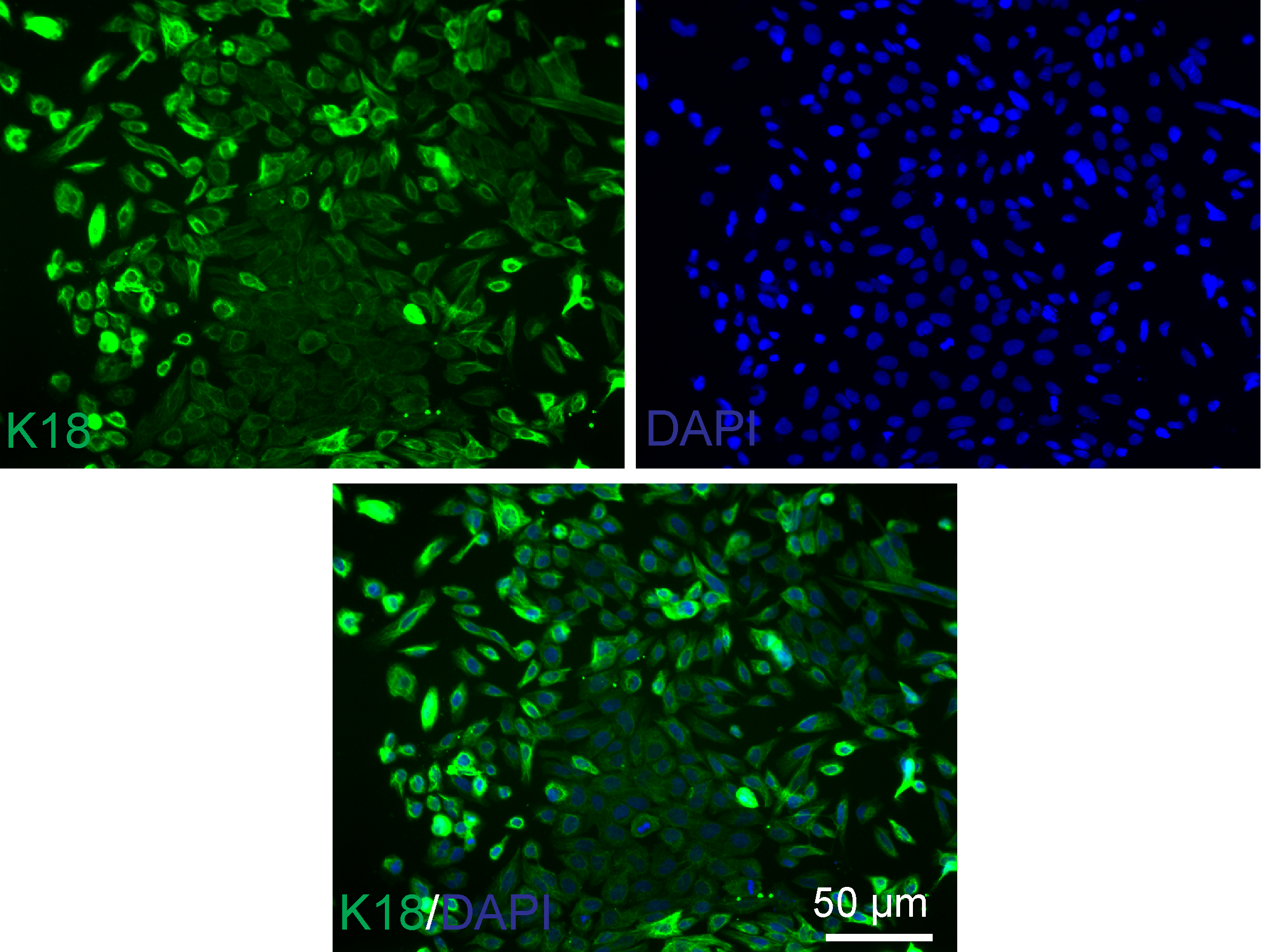

Supplement: Figure S3 — Keratin 18 expression in simple epithelial cells after two month expansion. Subculture of H9-derived simple epithelial cells (K18+) over 60 days. After 60 days of expansion, cells were fixed and immunostaining of K18 was performed. Representative K18 (green) immunocytochemistry and DAPI (blue) staining are shown. (TIF) [file pone.0060016.s003.tif]

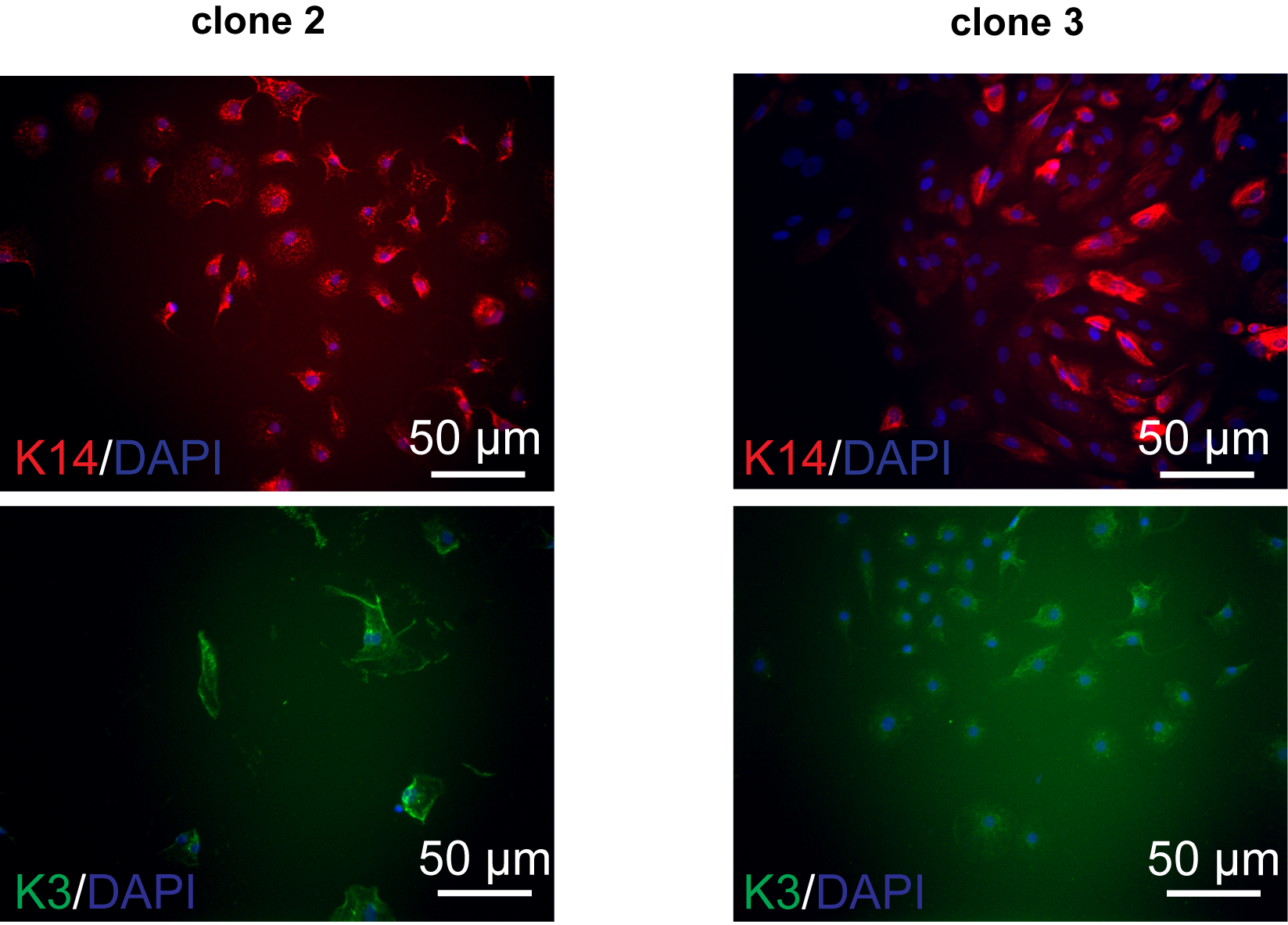

Supplement: Figure S4 — Single K18+ cell differentiation to K14+ and K3+ cells. Single K18+ cell-derived clones were manually picked and plated on Matrigel-coated 24-well plates at a density of one clone per well. Cells were treated with 1 µM RA for 4 days and then cultured with medium changes every 3 days for another 2 weeks before performing immunostaining with K14 (red) and K3 (green) antibodies. Scale bars = 50 µm. (TIF) [file pone.0060016.s004.tif]
